# A Dual PI3K/HDAC Inhibitor Downregulates Oncogenic Pathways in Hematologic Tumors *In Vitro* and *In Vivo*

**DOI:** 10.3389/fphar.2021.741697

**Published:** 2021-10-01

**Authors:** Zheng Yan, Kehui Zhang, Ming Ji, Heng Xu, Xiaoguang Chen

**Affiliations:** Institute of Materia Medica, Chinese Academy of Medical Sciences and Peking Union Medical College, Beijing, China

**Keywords:** dual inhibitor, PI3K, HDAC, ErbB2, ErbB3

## Abstract

Purpose: To investigate the efficacy and mechanism of compound 23, a PI3K/HDAC dual-target inhibitor, on hematologic tumor cells *in vitro* and *in vivo.* Methods: The MTS Kit was used to study the antiproliferative effects *in vitro*. Western blot was used to analyze the involved signaling pathways. Flow cytometry was used to analyze apoptosis and the cell cycle. The antiproliferative effects were evaluated *in vivo* using EL4 and A20 xenograft models. The CCLE database was used to analyze gene expression. Results: Compound **23** significantly inhibited the proliferation of hematologic tumors; it simultaneously regulated PI3K/HDAC pathways and induced apoptosis and G1-phase arrest in EL4, NB4, and A20 cells *in vitro*. When tested *in vivo*, compound **23** significantly inhibited the proliferation of EL4 and A20. The expression levels of ErbB2 and ErbB3 decreased in hematologic tumors compared with it in solid tumors. Conclusion: Compound **23** modulates the PI3K/HDAC pathway, which results in significant inhibition of hematologic tumor proliferation *in vivo* and *in vitro.* The differential levels of ERBB2 and ERBB3 might be related to the difference in the effect of compound **23** on hematologic tumors and solid tumors.

## Introduction

Polypharmacological drugs can simultaneously and specifically modulate multiple targets (de Lera and Ganesan, 2016) to produce additive or synergistic effects while reducing side effects (Proschak et al., 2019). Administration of such drugs is of particular use for diseases with complex mechanisms, such as cancer and central nervous system disorders (Anighoro et al., 2014). The discoveries of sorafenib (the first multikinase inhibitor of Raf isoforms) and inhibitors of receptor tyrosine kinases (RTKs: such as vascular endothelial growth factor receptors (Anighoro et al., 2014; de Lera and Ganesan, 2016; Proschak et al., 2019), platelet-derived growth factor receptor β, c-Kit, Flt-3, and RET) have been a landmark of targeted cancer therapy (Wilhelm et al., 2006). Class I phosphoinositide 3-kinases (PI3Ks), members of the PI3K family, are lipid kinases and are involved in multiple cellular processes, including proliferation, differentiation, migration, metabolism, and survival (Engelman et al., 2006). In addition, Class I PI3Ks have been one of the most intensively pursued targets for therapeutic intervention in cancer (Cantley, 2002; Liu et al., 2009; Janku et al., 2018). The PI3K pathway is activated by cell surface receptors, such as RTKs or G protein-coupled receptors, resulting in recruitment of class IA PI3Ks to the cell membrane, whereby they convert phosphatidylinositol-4,5-bisphosphate to phosphatidylinositol-3,4,5-trisphosphate. Simultaneously, activation of the Ras/Raf/mitogen-activated protein kinase (MAPK) pathway can lead to a crosstalk with PI3K (Sarbassov et al., 2005; Yip, 2015). Recent comprehensive cancer genomic analyses revealed that multiple components of the PI3K pathway are frequently mutated or altered in common human cancers (Samuels et al., 2004; Thomas et al., 2007; Wood et al., 2007; Cancer Genome Atlas Research Network, 2008; Parsons et al., 2008). To date, three PI3K inhibitors, idelalisib (Miller et al., 2015), copanlisib (Markham, 2017), and duvelisib (Blair, 2018), have been approved by the United States Food and Drug Administration (FDA) for the treatment of hematologic cancers.

Histone deacetylases (HDACs)—critical regulators of chromatin conformation and transcription—enzymatically remove the acetyl group from histone and non-histone proteins (West and Johnstone, 2014). Abnormal HDACs have been documented to play key roles in many human diseases including (but not limited to) cancer, neurological diseases, metabolic disorders, inflammatory diseases, cardiac diseases, and pulmonary diseases (Seto and Yoshida, 2014; Zhang et al., 2018). HDAC inhibitors (HDACis) can induce tumor cell apoptosis, growth arrest, senescence, differentiation, and immunogenicity, and they also have the capacity to inhibit angiogenesis (Minucci and Pelicci, 2006; West and Johnstone, 2014). To date, the following four HDAC inhibitors have been approved by the FDA for treatment of hematologic cancers: vorinostat, romidepsin, belinostat, and panobinostat (West and Johnstone, 2014; Shi et al., 2015; Manal et al., 2016; Yoon and Eom, 2016).

**FIGURE 1:**
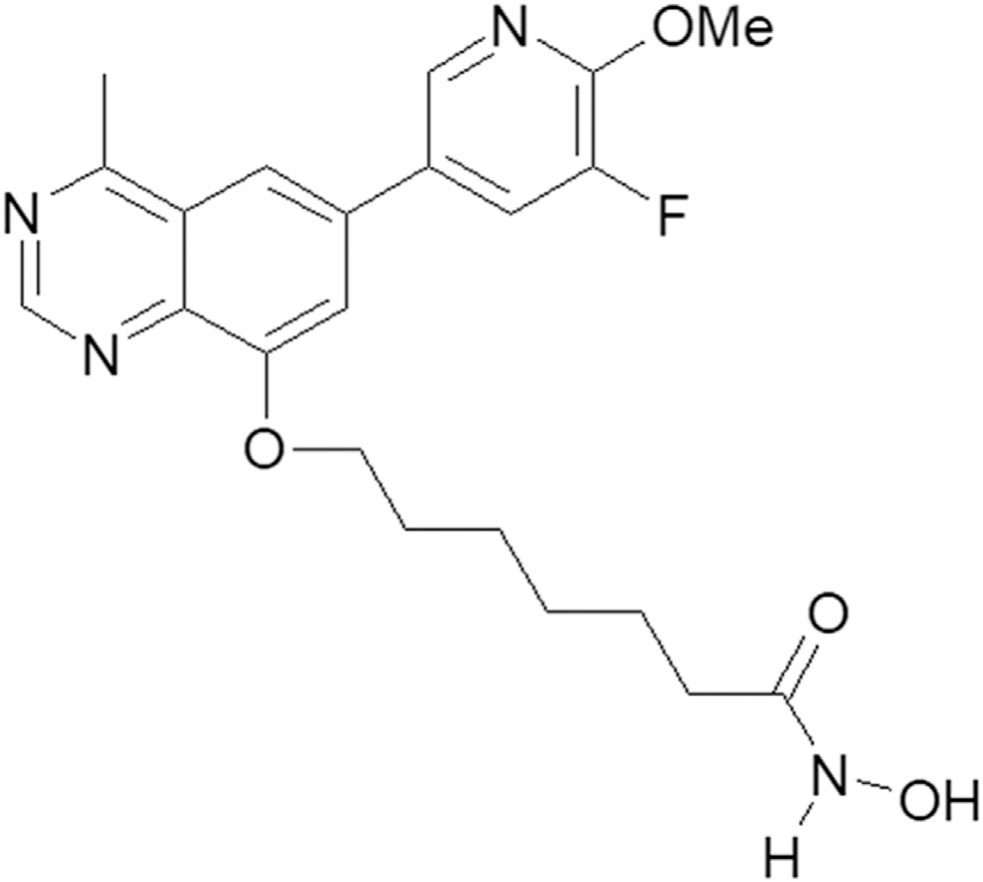
The structure of compound 23.

The HDAC pharmacophore acquires its inhibitory activity from the incorporation of a zinc-binding group (e.g., hydroxamic acid) through a linker (Zhang et al., 2018). The structure of the PI3K pharmacophore is based on a quinazoline skeleton. A linker has been adopted to integrate PI3K and HDAC pharmacophores into a PI3K/HDAC dual-targeting inhibitor (Rodrigues et al., 2020; Thakur et al., 2020). Based on previous studies of the structure–activity relationship, the structure of compound **23** includes a linker composed of six alkyls and quinazoline substituted with fluorine at position 8 (Figure 1). Through the induction of apoptosis and cell-cycle arrest in the G1 phase, compound **23** simultaneously regulates PI3K and HDAC signaling pathways, thereby exerting significant antiproliferative effects on solid tumor cells *in vitro* and *in vivo* (Zhang et al., 2019). Most therapeutic strategies for hematologic cancers, including T-cell acute lymphoblastic leukemia (Hales et al., 2014), acute myeloid leukemia (Li et al., 2019), chronic lymphocytic leukemia, small lymphocytic lymphoma (Blair, 2018), diffuse large B-cell lymphoma (DLBCL) (Kalac et al., 2011), cutaneous T-cell lymphoma (West and Johnstone, 2014), and others, involve regulation of PI3K/AKT/mechanistic target of rapamycin (mTOR) and HDAC pathways (Yoon and Eom, 2016). Therefore, on the basis of our previous work, compound **23** was investigated to assess its efficacy and mechanism of action against hematologic tumors.

## Materials and Methods

### Cell Viability Assay

*In vitro*, inhibitory effects of compound **23** were measured by a 3-(4,5-dimethylthiazol-2-yl)-5-(3-carboxymethoxyphenyl)-2-(4-sulfophenyl)-2H-tetrazolium) (MTS) assay. Briefly, cells were placed in 96-well plates at a density of 2 × 10^4^ cells/well. After equilibration overnight at 37°C, the cells were treated with compounds at various concentrations. After 72 h of culture, the MTS reagent was added to each well, and the plates were incubated for another 4 h at 37°C. The samples were measured using a micro-plate reader at a wavelength of 490 nm (Biotek Instruments, Winooski, VT, United States). Half-maximal inhibitory concentration (IC_50_) values were calculated with SigmaPlot 8.0 software (Systat Software, San Jose, CA, United States).

### Flow Cytometry Assay (Cell Cycle and Apoptosis)

The cell cycle and apoptosis were analyzed by flow cytometry. EL4, NB4, and A20 cells (2×10^6^ cells/well) were placed in 24-well plates. After equilibration overnight, the cells were treated with various concentrations of compounds for 24 h. The effects of the compounds on cell cycle progression and apoptosis were determined by flow cytometry analysis. Procedures were conducted in accordance with the manufacturer’s recommendations. FxCycle™ PI/RNase Staining Solution for cell cycle analysis was purchased from Thermo Fisher Scientific (Waltham, MA, United States). An Annexin V-FITC/PI Apoptosis Detection Kit was purchased from Beijing Kang Run Cheng Ye Biotech (Beijing, China).

### Western Blot

EL4, NB4, and A20 cells were collected and washed twice with cold phosphate-buffered saline (PBS) before being lysed with radioimmunoprecipitation assay buffer for protein isolation. Protein samples (40 μg) were separated by 10–12% sodium dodecyl sulfate polyacrylamide gel electrophoresis and then transferred to PVDF membranes by wet electrophoresis. The membranes were blocked with 5% milk in Tris-buffered saline containing Tween 20 (TBST) for 30 min, followed by incubation overnight with primary antibodies (1:1,000 dilution) at 4°C. After washing three times with TBST, the membranes were incubated with secondary antibodies (1:1,000) for 1 h. The resulting bands were visualized using an enhanced chemiluminescence detection kit and Image Quant LAS 4000 (GE Healthcare, Piscataway, NJ, United States).

### *In Vivo* Studies

After culture and harvesting, the cells were suspended in saline at a density of 2 × 10^7^ cells/mL. The animal protocol followed guidelines and was approved by the Experimental Animal Management and Welfare Committee at the Institute of Materia Medica, Peking Union Medical College (Beijing, China). Male mice (BALB/c C57 and BALB/c) aged 6–8 weeks were subcutaneously injected in their right flanks with 0.2 ml of EL4 or A20 cell solution. The EL4- and A20-injected mice were then randomly divided into five groups: a control group that received saline intraperitoneally (i.p.) every day; three groups that received compound 23 dissolved in saline at doses of 3.75, 7.5, and 15 mg/kg intraperitoneally; and a positive control group that received 100 mg/kg of suberoylanilide hydroxamic (SAHA) orally. Body weight of the mice was measured twice each week. After sacrificing the mice, tumors were isolated and weighed. The percentage of tumor weight inhibition (TWI%) was calculated as follows: TWI %= (1—Tumor Weight treatment/Tumor Weight vehicle) × 100%. Statistical analysis was performed in EXCEL GraphPad Prism 8.0 (GraphPad Software, San Diego, CA, United States), and the significance level was evaluated with a two-way Analysis of Variance (ANOVA) model.

### CCLE Analysis

The expression levels of PI3Kα (ENG00000121879), Akt (ENG00000142208), S6 (ENG00000108443), HDAC1 (ENG00000116478), TuBulin1α (ENG00000167552), ERBB2 (ENSG00000141736), and ERBB3 (ENSG00000065361) in different cell lines were analyzed by Cancer Cell Line Encyclopedia (CCLE, https://portals. broadinstitute. org/ccle), a database offering the expression level sorting of 84,434 genes in 1,457 cancer cell lines.

## Results

### Compound 23, a Dual HDAC and PI3K Inhibitor, Significantly Decreases Hematologic Cells Viability

A series of dual HDAC and PI3K inhibitors were synthesized to examine their antitumor efficacy. Among those compounds, compound **23** showed more potent antiproliferative activity against solid tumors. The fact that HDAC and PI3K inhibitors had been approved by the FDA for treatment of hematologic cancers encouraged us to test the antiproliferative activity of compound **23** against hematologic tumor cells. *In vitro* antiproliferative activity of compound **23** was evaluated by the MTS assay in hematologic tumor cells including T lymphoma (EL4), B lymphoma (A20), and leukemia (NB4). As shown in Table 1, compound **23** displayed potent antiproliferative activity compared with reference compounds (**SAHA** and **BKM120**). Notably, compound **23** showed more remarkable antiproliferative activity in hematologic cell lines (IC_50_ = 0.016–0.34 μM) compared with solid cancer cell lines (IC_50_ = 0.11–6.8 μM) (Zhang et al., 2019).

**TABLE 1:** Antiproliferative activity of compound **23** against various hematologic cell lines compared with **SAHA** and **BKM120**.

Cell types	Cell lines	IC_50_ (μM) in growth inhibition assay		
		SAHA	BKM120	23
Leukemia	K562	3.95 ± 1.47	1.19 ± 0.04	0.34 ± 0.27
Leukemia	HL60	0.88 ± 0.01	0.27 ± 0.01	0.052 ± 0.031
Leukemia	U937	1.68 ± 0.12	0.19 ± 0.15	0.071 ± 0.03
Leukemia	L1210	11.77 ± 1.95	4.11 ± 0.50	0.93 ± 0.04
Leukemia	NB4	15.40 ± 0.17	0.90 ± 0.06	0.02 ± 0.01
T lymphocyte	EL4	3.67 ± 1.79	2.37 ± 0.21	0.27 ± 0.11
T lymphocyte	Jurkat	1.69 ± 1.21	0.69 ± 0.13	0.034 ± 0.012
T lymphocyte	Yac-1	0.39 ± 0.04	0.74 ± 0.01	0.06 ± 0.02
B lymphocyte	Ramos	1.52 ± 0.34	0.44 ± 0.12	0.14 ± 0.04
B lymphocyte	BAF-3	0.199 ± 0.004	1.35 ± 0.09	0.023 ± 0.001
B lymphocyte	A20	0.67 ± 0.15	2.92 ± 0.72	0.07 ± 0.02

### Compound 23 Modulates HDAC and PI3K/mTOR Pathways in EL4, NB4, and A20 Cells

**FIGURE 2:**
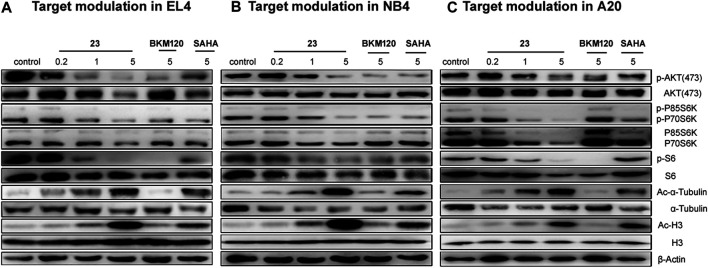
Modulation of the PI3K/AKT/mTOR and HDAC pathways in three hematologic tumor cell lines. **(A)** EL4, **(B)** NB4, and **(C)** A20 cells were treated with several concentrations of compound **23** for 24 h. Whole cell lysates were subjected to western blotting.

In our previous study, compound **23** displayed selective inhibition of HDAC and PI3 kinase (Zhang et al., 2019). Therefore, western blot was applied to study the effects of compound **23** on PI3K and HDAC pathways. According to the literature, the inhibition of HDAC induced hyperacetylation of histone and its other substrates such as α-tubulin, which upregulated acetylation of H3 and α-tubulin; the inhibition of the PI3K/AKT/mTOR pathway downregulated phosphorylation levels of proteins downstream of AKT and mTOR, such as P70S6K, P85S6K, and S6. After treatment with compound **23**, **BKM120**, or **SAHA** for 24 h, the three hematologic tumor cell lines were collected and fully lysed, and then the cell lysates were analyzed by western blot. As shown in Figure 2, compound **23** dose-dependently and simultaneously downregulated the levels of phosphor-AKT (p-AKT), phospho-p70 S6 kinase (p-P70 S6K), and phosphor-S6 ribosomal protein (p-S6), and upregulated acetyl histone H3 (Lys9) (Ac-H3) and acetyl-α-tubulin (Ac-α-tubulin) in the three cell lines. Moreover, compound **23** was more potent than **BKM120** and **SAHA** in terms of downregulation of phosphorylation and upregulation of acetylation, respectively.

### Compound 23 Significantly Increases the Proportion of Apoptosis in EL4, NB4, and A20 Cells

**FIGURE 3:**
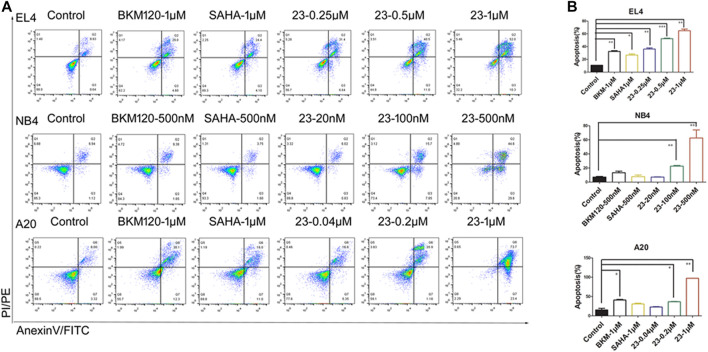
Cell apoptosis induced by compound **23** (**23**). EL4, NB4, and A20 cells were treated by the indicated concentration of compound **23**, **SAHA**, and **BKM120** for 24 h. Cells incubated in DMSO were used for comparison. **(A)** Representative histograms of Annexin V-FITC *vs* propidium iodide (PI)-PE staining results of flow cytometry. **(B)** Percentages of apoptotic cells (including early and late apoptotic cells) analyzed by flow cytometry (*p < 0.05, **p < 0.01, ***p < 0.001 vs. Control).

Recent studies have shown that HDAC and PI3K inhibitors can induce cell apoptosis (Chen et al., 2018). Since our previous results had shown that compound **23** significantly induced apoptosis in solid tumors (Zhang et al., 2019), we studied the proapoptotic effect of compound **23** in hematologic tumor cell lines, including EL4, NB4, and A20 cells. Annexin-V and propidium iodide (PI) staining were performed to investigate the effect on apoptosis. As shown in Figure 3, compound **23** significantly increased the proportions of early and late apoptotic cells of all three tumor cell lines in a dose-dependent manner and more potently induced apoptosis compared with **BKM120** and **SAHA** at the same dose. Furthermore, it was remarkable that the apoptosis rates of EL4, NB4, and A20 cells treated with compound **23** at 500 μM were 52.37%, 62.65%, and more than 36.48%, respectively, which were both superior to the apoptosis rates (less than 30%) of HCT116 treated by compound **23** at the same dose (Zhang et al., 2019). Compared to the previous data, compound **23** has a more potent proapoptotic effect on hematologic tumors.

### Compound 23 Causes G1 Arrest in EL4, NB4, and A20 Cells

**FIGURE 4:**
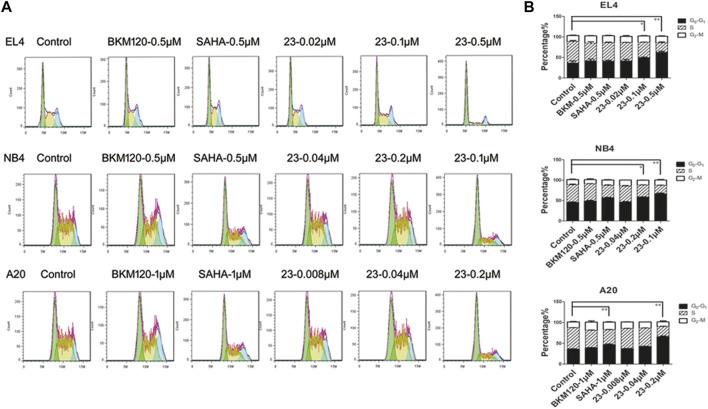
Compound **23** induced G1-phase cell cycle arrest. EL4, NB4, and A20 cells were treated with dimethyl sulfoxide (DMSO), **SAHA**, **BKM120**, or DMSO with increasing concentrations of **23** for 24 h. Flow cytometry analysis of propidium iodide (PI) staining was used to assess cell cycle distribution. **(A)** Representative histograms of cell cycle distribution analyzed by PI staining. **(B)** Bar columns showing relative percentages of cells in S, G1, and G2 phases of the cell cycle following treatment with compound **23** or reference compounds (*p < 0.05, **p < 0.01, ***p < 0.001 vs Control).

Since both HDAC and PI3K pathways are involved in cell processes such as proliferation, differentiation, and migration, here we evaluated cell cycle distribution to investigate the influence of compound **23** on hematologic tumor cells. As shown in Figure 4, histograms were prepared to demonstrate the influence on the cell cycle. Compound **23** arrested the cell cycle in G1 phase in all three cell lines. We observed that compound **23** and **SAHA** caused a significant cell cycle arrest at G1 phase in all three cell types. The proportions of cells in G1 phase in the control groups of EL4, NB4, and A20 cell lines were 36.7, 45.5, and 36.3%, respectively; in contrast, the proportions of G1 phase-arrested cells of EL4, NB4, and A20 cell lines significantly increased to 63, 66.5, and 65.8% in the compound **23** high-dose group. Conversely, **BKM120** had no significant effect on the cell cycle. According to our results, compound **23** was able to arrest the cell cycle in G1 in different hematologic tumor cell lines.

### Compound 23 Contributes to Tumor Growth Inhibition in a T Lymphoma Xenograft Model

**FIGURE 5:**
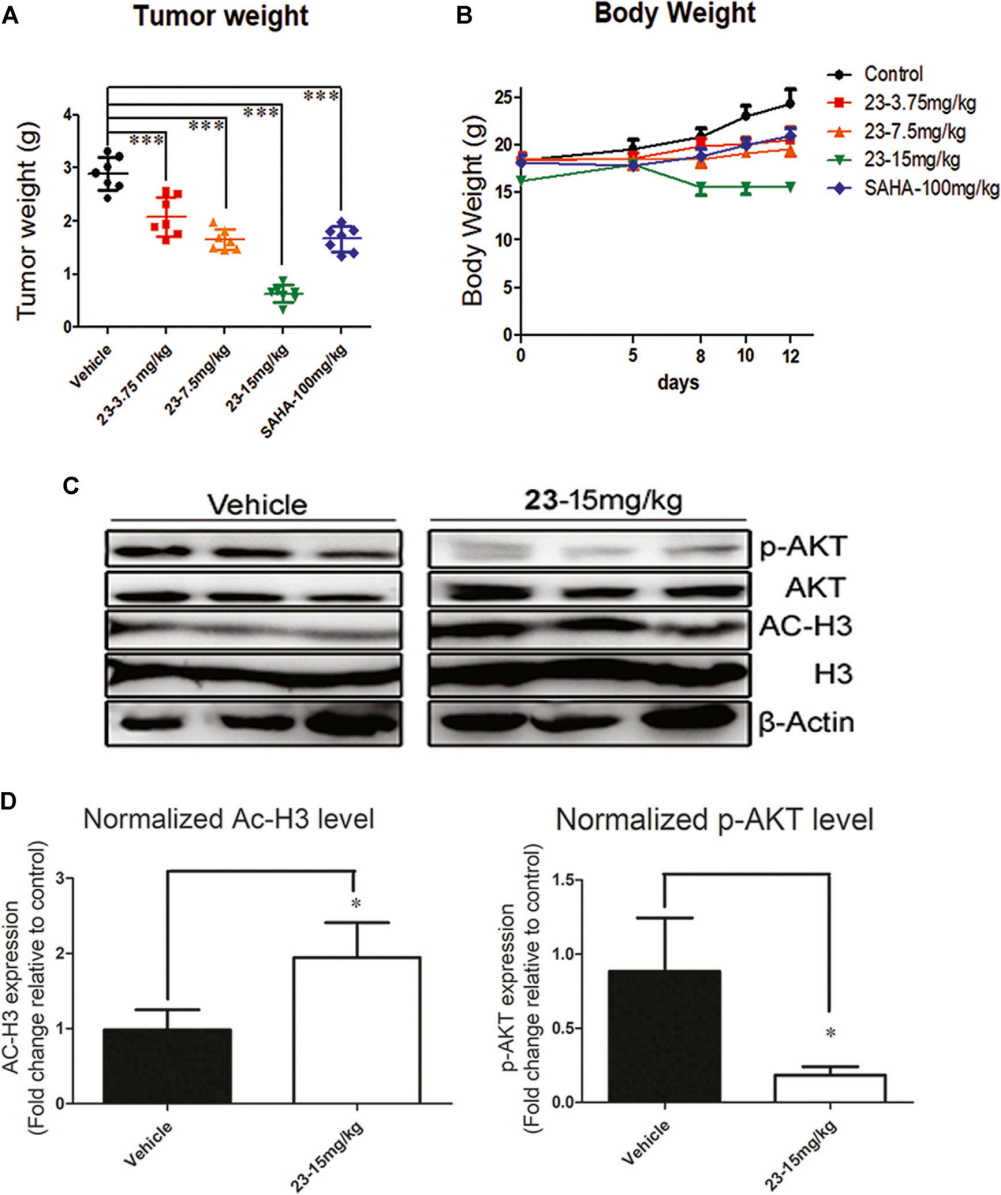
An EL4 xenograft model was included to evaluate the antitumor activity of compound 23 against T lymphoma *in vivo*. **(A)**. Tumor weight after intraperitoneal administration of compound **23** at dosages of 3.75, 7.5 and 15mg/kg or oral administration of SAHA at dosage of 100mg/kg. **(B)**. Average body weight changes of compound **23**, **SAHA**, and vehicle groups during administration. **(C)** and **(D)**. The modulation on HDAC and AKT pathways in EL4 tissue in 15 mg/kg of compound **23** group and vehicle group (*p < 0.05, **p < 0.01, ***p < 0.001 vs. Control).

Compound **23** inhibited the variation of hematologic cells, induced apoptosis, and promoted cell arrest *in vitro*. Therefore, we further assessed the antitumor potency of compound **23** against T lymphoma and B lymphoma *in vivo*. After 12 days of administration, the tumor tissues were isolated and weighed. The TWI% of compound **23** at 3.75 mg/kg, 7.5 mg/kg, and 15 mg/kg was 27.8, 42.9, and 78.1%, respectively (Figure 5A). As shown in Figure 5B, although compound **23** induced weight loss in animals during administration, it still significantly inhibited tumor growth in the high-dose group compared with both the vehicle and positive control groups. The tumor tissues from three tumor-bearing mice in each group of vehicle and compound **23** at 15 mg/kg group were randomly selected, and their supernatants were analyzed by western blot after full lysis. Compound 23 exhibited more potent antitumor activity compared with **SAHA** by simultaneously regulating PI3K and HADAC signaling pathways *in vivo* as shown in Figures 5C,D.

### Compound 23 Contributes to Tumor Growth Inhibition in a B Lymphoma Xenograft Model

**FIGURE 6:**
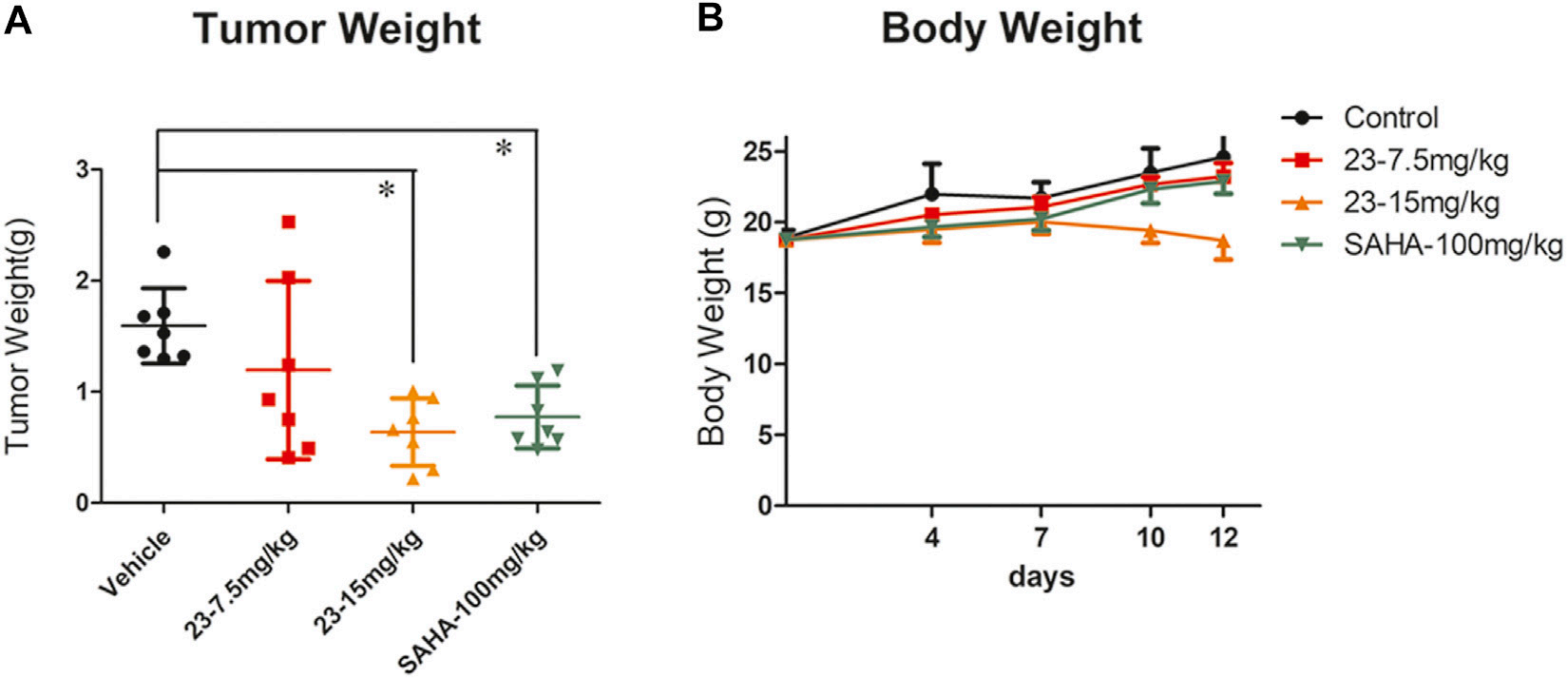
An A20 xenograft model was included to evaluate the antitumor activity of compound **23** against B lymphoma *in vivo*. **(A)** Tumor weight after intraperitoneal administration of compound **23** at dosages of 7.5 and 15 mg/kg, or oral administration of **SAHA** at a dosage of 100 mg/kg. **(B)** Average body weight changes of compound **23**, **SAHA**, and vehicle groups during administration (*p < 0.05, **p < 0.01, ***p < 0.001 vs. Control).

After confirming that compound **23** significantly inhibited the proliferation of mouse T lymphoma *in vivo*, we also explored whether it had inhibitory activity against B lymphoma. The TWI% of compound **23** at 7.5 mg/kg and 15 mg/kg was 24.9 and 60%, respectively (Figure 6A). Although compound **23** showed some toxicity by reducing body weight in animals at 15 mg/kg, it still significantly inhibited the proliferation of B lymphoma. In addition, it showed stronger antitumor activity than **SAHA** at 15 mg/kg *in vivo* (Figure 6B).

### Hematologic Tumor Cell Lines Express Lower ErbB2 and ErbB3 Compared With Solid Tumors

**FIGURE 7:**
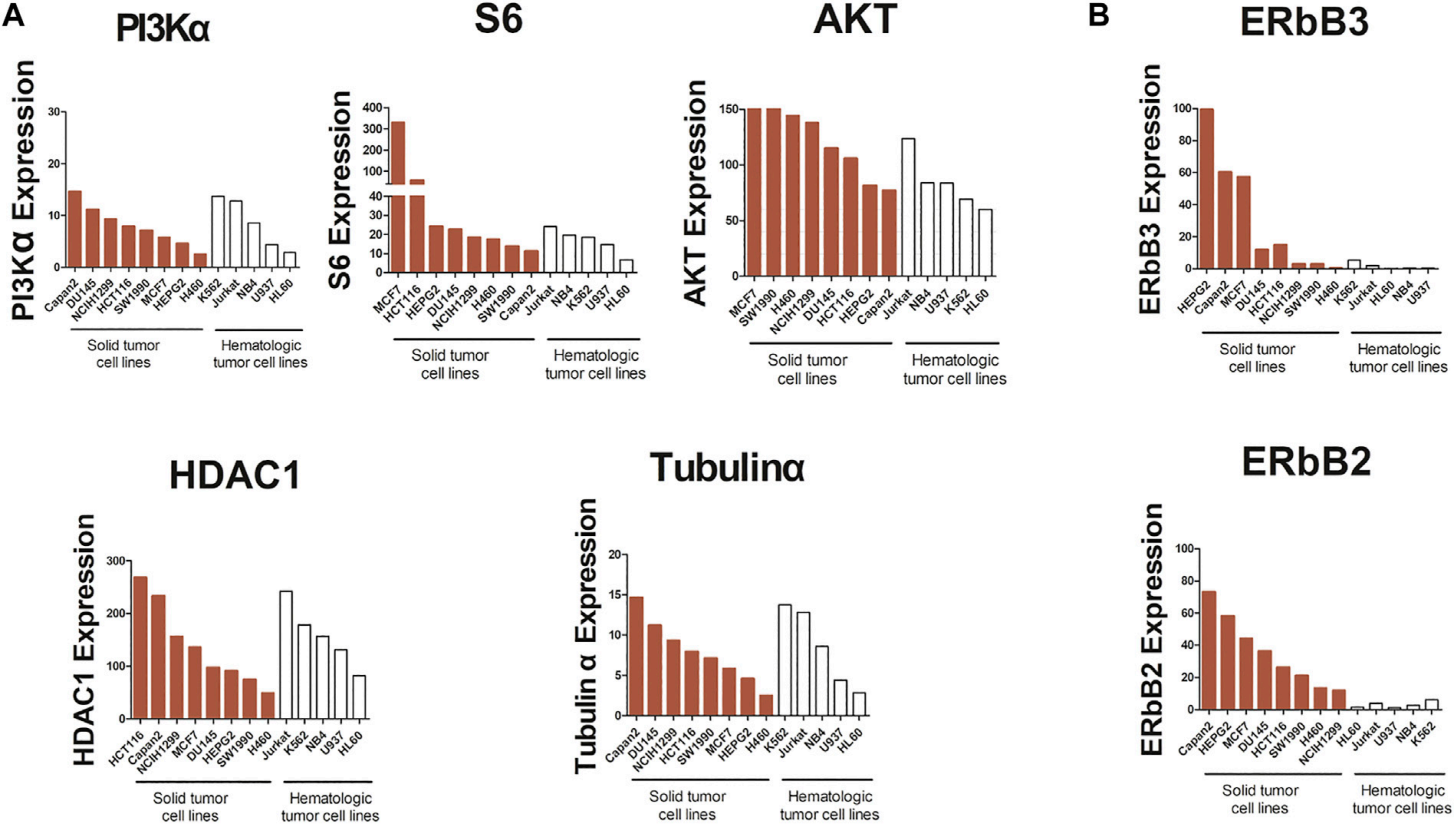
Analysis of PI3Kα, S6, AKT, HDAC1, Tubulinα, ERbB3, and ERbB2 mRNA expression in cancer cells. **(A)** The expression of PI3Kα, S6, AKT, HDAC1 and Tubulinα in solid tumors and hematologic tumors at mRNA level. **(B)** The expression of ERbB2 and ERbB3 in solid tumors and hematologic tumors at mRNA level.

Comparing with the results of previous studies, we found that compound **23** showed a more potent antitumor activity against hematologic tumors compared with solid tumors. Many cellular processes in cancer are attributed to kinase signaling networks (Song et al., 2019). To understand the reasons for the discrepancy of the effects of compound **23** between solid tumors and hematologic tumors, we identified the gene expression levels of kinases in the PI3K/Akt/mTOR axis and HDAC pathway, such as PI3Kα, Akt, S6, HDAC1, and TuBulin1α, through the CCLE database (https://portals.broadinstitute.org). As shown in Figure 7A, we observed an analogous pattern of expression of the aforementioned kinases in both signaling pathways between solid tumors and hematologic tumors at the gene level. However, as shown in Figure 7B, the expression levels of ERBB2 and ERBB3 decreased in hematologic tumors compared with it in solid tumors. The overexpression of Erb2B in numerous types of primary human tumors and alterations in microRNA (miRNA) have been associated with tumor suppression or tumorigenesis in human cancer (Chen et al., 2009). Expression of certain oncogenes can result in activation of the PI3K/AKT signaling pathway. ErbB2 and ErbB3 form heterodimers that could activate the downstream targets of the PI3K/Akt signaling pathway (Hong et al., 2021). Thus, the low expression of ErbB2 and ErbB3 stabilized the PI3K/Akt signaling pathway in hematologic tumors (Table 1), thereby enabling compound **23** to exhibit more potent antitumor efficacy than against solid tumor cells. Based on these data, the expression levels of ErbB2 and ErbB3 at the gene level might be related to the effect of compound **23** and even the therapeutic effect of this type of compound, which may provide some reference value for the response rate of compound **23** in clinical treatment.

## Discussion

Simply bridging two pharmacophores through a linker often does not work out for dual-target designs. The purpose of our design of dual-target compounds is to achieve an optimal balance of activity of two pharmacophores. So, we previously synthesized a series of compounds. Among them, compound **23**, which adopted a six alkyls linker and fluorine substituted in position 8 of quinazoline, displayed simultaneous regulatory effects on PI3K and HDAC signaling pathways, antiproliferative effects on solid cancer cells *in vitro* and *in vivo*, and induction of apoptosis and cycle arrest.

Having been granted fast-track designation by the FDA for treatment of relapsed or refractory DLBCL after positive results from phase II studies (CURIS, 2019), the first PI3K/HDAC dual inhibitor **GUDC-907** was investigated in clinical trials, which inspired us to observe the antitumor activity of compound **23** against hematologic tumors. Eleven hematologic tumor cells, including T lymphoma, B lymphoma, and leukemia cells, were examined to evaluate the antitumor activity of compound **23** *in vitro.* According to our results, compound **23** demonstrated more potent antitumor activity relative to the reference compound, and the efficacy against hematologic tumors was superior to that against solid tumors. Sequentially, we observed the regulatory effect of compound **23** on PI3K and HDAC signaling pathways. The PI3K/AKT/mTOR axis is one of the most frequently dysregulated pathways in cancer (Janku et al., 2018). The activation of the PI3K signaling pathway contributes to cell proliferation, survival, and motility, as well as angiogenesis, all of which are responsible for the important aspects of tumorigenesis (Liu et al., 2009). HDACs play a vital role in epigenetic regulation of gene expression. Due to their aberrant activity and overexpression in several forms of cancer, HDACs are considered to be a potential anticancer drug target (Manal et al., 2016). Three cell lines, EL4, NB4, and A20, were used to evaluate the role of compound **23** in regulating the two signaling pathways through western blot. We examined the levels of acetylation and phosphorylation of a series of kinases in the two signaling pathways. The results showed that compound **23** simultaneously regulated PI3K and HDAC signaling pathways by upregulating Ac-H3 and Ac-tubulin and downregulating p-Akt (S473), p-P70S6K, p-P80S6K, and p-S6 in a dose-dependent manner; moreover, the regulatory effect of compound **23** was stronger than that of **SAHA** and **BKM120**. Since the PI3K and HDAC signaling pathways are involved in cell proliferation, we investigated the effects of compound **23** on apoptosis and cycle arrest in the three hematologic tumor cells mentioned above. The results indicated that compound **23** significantly inhibited the proliferation of hematologic tumor cells, while it simultaneously induced apoptosis and G1 phase arrest in a dose-dependent manner compared with the reference compounds **SAHA** and **BKM120**; these effects on hematologic tumor cycle arrest and apoptosis were more potent than those on solid tumors. According to the previous study of compound **23** in a solid tumor xenograft model, the TWIs of compound **23** to the solid tumor xenograft model, HCT116 and HCG27, were 45.8 and 62.6%, respectively, at 150 mg/kg and 30 mg/kg intraperitoneally administered doses (Zhang et al., 2019). The TWIs of compound **23** to the hematologic tumor xenograft model, EL4 and A20, were 78.1 and 60% at 15 mg/kg, respectively, indicating that compound **23** obtained superior efficacy against hematologic tumors at a lower dosage compared with its efficacy against solid tumors. We found that compound **23** was more effective against hematologic tumors than against solid tumors. In an attempt to understand the reasons for the differential efficacy between solid tumors and hematologic tumors, we conducted bioinformatics analysis. By detecting the expression of kinases at the gene level in the PI3K/HDAC pathways with the CCLE database, we found that the expression levels of ErbB2 and ErbB3 in hematologic tumor cells were lower than those in solid tumors. Therefore, we suggest that the efficacy of compound 23 against hematologic tumors may be related to the expression levels of ErbB2 and ErbB3 in hematologic tumor cells.

In summary, compound **23**, with a six alkyls linker and fluorine iodide in position 8 of quinazoline, showed its synergistic effect by simultaneously regulating PI3K and HDAC signaling pathways to significantly inhibit the proliferation of hematologic tumor cells and induce apoptosis and G1 arrest *in vivo* or *in vitro*.

## Nomenclature

### Resource Identification Initiative

To take part in the Resource Identification Initiative, please use the corresponding catalog number and RRID in your current manuscript. For more information about the project and for steps on how to search for an RRID, please click here.

### Life Science Identifiers

Life Science Identifiers (LSIDs) for ZOOBANK registered names or nomenclatural acts should be listed in the manuscript before the keywords with the following format:

urn:lsid:<Authority>:<Namespace>:<ObjectID>[:<Version>]

For more information on LSIDs please see Inclusion of Zoological Nomenclature section of the guidelines.

## Data Availability

The original contributions presented in the study are included in the article/supplementary material, further inquiries can be directed to the corresponding author.

## References

[B1] Anighoro A. Bajorath J. Rastelli G. (2014). Polypharmacology: Challenges and Opportunities in Drug Discovery. J. Med. Chem. 57 (19), 7874–7887. 10.1021/jm5006463 24946140

[B2] Blair H. A. (2018). Duvelisib: First Global Approval. Drugs 78 (17), 1847–1853. 10.1007/s40265-018-1013-4 30430368

[B3] Cancer Genome Atlas Research Network (2008). Comprehensive Genomic Characterization Defines Human Glioblastoma Genes and Core Pathways. Nature 455 (7216), 1061–1068. 10.1038/nature07385 18772890 PMC2671642

[B4] Cantley L. C. (2002). The Phosphoinositide 3-kinase Pathway. Science 296 (5573), 1655–1657. 10.1126/science.296.5573.1655 12040186

[B5] Chen D. Soh C. K. Goh W. H. Wang H. (2018). Design, Synthesis, and Preclinical Evaluation of Fused Pyrimidine-Based Hydroxamates for the Treatment of Hepatocellular Carcinoma. J. Med. Chem. 61 (4), 1552–1575. 10.1021/acs.jmedchem.7b01465 29360358

[B6] Chen H. Sun J. G. Cao X. W. Ma X. G. Xu J. P. Luo F. K. (2009). Preliminary Validation of ERBB2 Expression Regulated by miR-548d-3p and miR-559. Biochem. Biophys. Res. Commun. 385 (4), 596–600. 10.1016/j.bbrc.2009.05.113 19486885

[B7] CURIS (2019). FIMEPINOSTAT: First-In-Class Suppressor of MYC. Available at: http://www.curis.com/pipeline/fimepinostat (Accessed Mar 3, 2019)

[B8] de Lera A. R. Ganesan A. (2016). Epigenetic Polypharmacology: from Combination Therapy to Multitargeted Drugs. Clin. Epigenetics 8, 105. 10.1186/s13148-016-0271-9 27752293 PMC5062873

[B9] Engelman J. A. Luo J. Cantley L. C. (2006). The Evolution of Phosphatidylinositol 3-kinases as Regulators of Growth and Metabolism. Nat. Rev. Genet. 7 (8), 606–619. 10.1038/nrg1879 16847462

[B10] Hales E. C. Taub J. W. Matherly L. H. (2014). New Insights into Notch1 Regulation of the PI3K-AKT-mTOR1 Signaling axis: Targeted Therapy of γ-secretase Inhibitor Resistant T-Cell Acute Lymphoblastic Leukemia. Cell Signal 26 (1), 149–161. 10.1016/j.cellsig.2013.09.021 24140475

[B11] Hong M. Yoo Y. Kim M. Kim J. Y. Cha J. S. Choi M. K. (2021). A Novel Therapeutic Anti-ErbB3, ISU104 Exhibits Potent Antitumorigenic Activity by Inhibiting Ligand Binding and ErbB3 Heterodimerization. Mol. Cancer Ther. 20 (6), 1142–1152. 10.1158/1535-7163.MCT-20-0907 33782100

[B12] Janku F. Yap T. A. Meric-Bernstam F. (2018). Targeting the PI3K Pathway in Cancer: Are We Making Headway? Nat. Rev. Clin. Oncol. 15 (5), 273–291. 10.1038/nrclinonc.2018.28 29508857

[B13] Kalac M. Scotto L. Marchi E. Amengual J. Seshan V. E. Bhagat G. (2011). HDAC Inhibitors and Decitabine Are Highly Synergistic and Associated with Unique Gene-Expression and Epigenetic Profiles in Models of DLBCL. Blood 118 (20), 5506–5516. 10.1182/blood-2011-02-336891 21772049 PMC3217353

[B14] Li X. Su Y. Madlambayan G. Edwards H. Polin L. Kushner J. (2019). Antileukemic Activity and Mechanism of Action of the Novel PI3K and Histone Deacetylase Dual Inhibitor CUDC-907 in Acute Myeloid Leukemia. Haematologica 104 (11), 2225–2240. 10.3324/haematol.2018.201343 30819918 PMC6821619

[B15] Liu P. Cheng H. Roberts T. M. Zhao J. J. (2009). Targeting the Phosphoinositide 3-kinase Pathway in Cancer. Nat. Rev. Drug Discov. 8 (8), 627–644. 10.1038/nrd2926 19644473 PMC3142564

[B16] Manal M. Chandrasekar M. J. Gomathi Priya J. Nanjan M. J. (2016). Inhibitors of Histone Deacetylase as Antitumor Agents: A Critical Review. Bioorg. Chem. 67, 18–42. 10.1016/j.bioorg.2016.05.005 27239721

[B17] Markham A. (2017). Copanlisib: First Global Approval. Drugs 77 (18), 2057–2062. 10.1007/s40265-017-0838-6 29127587

[B18] Miller B. W. Przepiorka D. de Claro R. A. Lee K. Nie L. Simpson N. (2015). FDA Approval: Idelalisib Monotherapy for the Treatment of Patients with Follicular Lymphoma and Small Lymphocytic Lymphoma. Clin. Cancer Res. 21 (7), 1525–1529. 10.1158/1078-0432.CCR-14-2522 25645861

[B19] Minucci S. Pelicci P. G. (2006). Histone Deacetylase Inhibitors and the Promise of Epigenetic (And More) Treatments for Cancer. Nat. Rev. Cancer 6 (1), 38–51. 10.1038/nrc1779 16397526

[B20] Parsons D. W. Jones S. Zhang X. Lin J. C. Leary R. J. Angenendt P. (2008). An Integrated Genomic Analysis of Human Glioblastoma Multiforme. Science 321 (5897), 1807–1812. 10.1126/science.1164382 18772396 PMC2820389

[B21] Proschak E. Stark H. Merk D. (2019). Polypharmacology by Design: A Medicinal Chemist's Perspective on Multitargeting Compounds. J. Med. Chem. 62 (2), 420–444. 10.1021/acs.jmedchem.8b00760 30035545

[B22] Rodrigues D. A. Guerra F. S. Sagrillo F. S. de Sena M Pinheiro P. Alves M. A. Thota S. (2020). Design, Synthesis, and Pharmacological Evaluation of First-In-Class Multitarget N-Acylhydrazone Derivatives as Selective HDAC6/8 and PI3Kα Inhibitors. ChemMedChem 15, 539–551. 10.1002/cmdc.201900716 32022441

[B23] Samuels Y. Wang Z. Bardelli A. Silliman N. Ptak J. Szabo S. (2004). High Frequency of Mutations of the PIK3CA Gene in Human Cancers. Science 304 (5670), 554. 10.1126/science.1096502 15016963

[B24] Sarbassov D. D. Guertin D. A. Ali S. M. Sabatini D. M. (2005). Phosphorylation and Regulation of Akt/PKB by the Rictor-mTOR Complex. Science 307 (5712), 1098–1101. 10.1126/science.1106148 15718470

[B25] Seto E. Yoshida M. (2014). Erasers of Histone Acetylation: the Histone Deacetylase Enzymes. Cold Spring Harb Perspect. Biol. 6 (4), a018713. 10.1101/cshperspect.a018713 24691964 PMC3970420

[B26] Shi Y. Dong M. Hong X. Zhang W. Feng J. Zhu J. (2015). Results from a Multicenter, Open-Label, Pivotal Phase II Study of Chidamide in Relapsed or Refractory Peripheral T-Cell Lymphoma. Ann. Oncol. 26 (8), 1766–1771. 10.1093/annonc/mdv237 26105599

[B27] Song M. Bode A. M. Dong Z. Lee M. H. (2019). AKT as a Therapeutic Target for Cancer. Cancer Res. 79 (6), 1019–1031. 10.1158/0008-5472.CAN-18-2738 30808672

[B28] Thakur A. Tawa G. J. Henderson M. J. Danchik C. Liu S. Shah P. (2020). Design, Synthesis, and Biological Evaluation of Quinazolin-4-One-Based Hydroxamic Acids as Dual PI3K/HDAC Inhibitors. J. Med. Chem. 63 (8), 4256–4292. 10.1021/acs.jmedchem.0c00193 32212730 PMC7238858

[B29] Thomas R. K. Baker A. C. Debiasi R. M. Winckler W. Laframboise T. Lin W. M. (2007). High-throughput Oncogene Mutation Profiling in Human Cancer. Nat. Genet. 39 (3), 347–351. 10.1038/ng1975 17293865

[B30] West A. C. Johnstone R. W. (2014). New and Emerging HDAC Inhibitors for Cancer Treatment. J. Clin. Invest. 124 (1), 30–39. 10.1172/JCI69738 24382387 PMC3871231

[B31] Wilhelm S. Carter C. Lynch M. Lowinger T. Dumas J. Smith R. A. (2006). Discovery and Development of Sorafenib: a Multikinase Inhibitor for Treating Cancer. Nat. Rev. Drug Discov. 5 (10), 835–844. 10.1038/nrd2130 17016424

[B32] Wood L. D. Parsons D. W. Jones S. Lin J. Sjöblom T. Leary R. J. (2007). The Genomic Landscapes of Human Breast and Colorectal Cancers. Science 318 (5853), 1108–1113. 10.1126/science.1145720 17932254

[B33] Yip P. Y. (2015). Phosphatidylinositol 3-Kinase-AKT-Mammalian Target of Rapamycin (PI3K-Akt-mTOR) Signaling Pathway in Non-small Cell Lung Cancer. Transl. Lung Cancer Res. 4 (2), 165–176. 10.3978/j.issn.2218-6751.2015.01.04 25870799 PMC4384220

[B34] Yoon S. Eom G. H. (2016). HDAC and HDAC Inhibitor: From Cancer to Cardiovascular Diseases. Chonnam Med. J. 52 (1), 1–11. 10.4068/cmj.2016.52.1.1 26865995 PMC4742605

[B35] Zhang K. Lai F. Lin S. Ji M. Zhang J. Zhang Y. (2019). Design, Synthesis, and Biological Evaluation of 4-Methyl Quinazoline Derivatives as Anticancer Agents Simultaneously Targeting Phosphoinositide 3-Kinases and Histone Deacetylases. J. Med. Chem. 62 (15), 6992–7014. 10.1021/acs.jmedchem.9b00390 31117517

[B36] Zhang L. Zhang J. Jiang Q. Zhang L. Song W. (2018). Zinc Binding Groups for Histone Deacetylase Inhibitors. J. Enzyme Inhib. Med. Chem. 33 (1), 714–721. 10.1080/14756366.2017.1417274 29616828 PMC6009916

